# Searching for a Vanishing Fish: Development of an eDNA-Based Digital PCR Assay and Field Surveys for the Critically Endangered Chinese Bahaba

**DOI:** 10.3390/biology15201842

**Published:** 2026-10-14

**Authors:** Chang Pan, Shuai Zhang, Honghui Huang, Lin Yan, Ying Pei, Wencheng Zhao, Hao Li, Junwei Li, Min Li

**Affiliations:** 1 South China Sea Fisheries Research Institute, Chinese Academy of Fishery Sciences, Guangzhou 510300, China; panchang@scsfri.ac.cn (C.P.);; 2 Guangdong Beluga Whale Marine Biotechnology Co., Ltd., Huizhou 516300, China; 3 Department of Infectious Diseases and Public Health, City University of Hong Kong, Kowloon Tong, Hong Kong SAR, China; 4 Guangdong Provincial Observation and Research Station for Ecosystem in the Pearl River Estuary, Guangzhou 510300, China; 5 Key Laboratory for Sustainable Utilization of Open-Sea Fishery, Ministry of Agriculture and Rural Affairs, Guangzhou 510300, China

**Keywords:** *Bahaba taipingensis*, environmental DNA (eDNA), digital PCR (dPCR), endangered species, Pearl River Estuary

## Abstract

The critically endangered Chinese bahaba (*Bahaba taipingensis*) is difficult to monitor via conventional methods due to its low abundance and estuarine habitat. We developed a species-specific microfluidic digital PCR assay targeting the mitochondrial *COI* gene for environmental DNA (eDNA) detection. No cross-amplification was observed with seven sympatric non-target sciaenid species. The limit of quantification (LOQ) was 1.313 copies µL^−1^ and the limit of detection (LOD) was bounded between 0.162 and 1.313 copies µL^−1^ among the tested concentrations. All 12 aquaculture samples yielded quantifiable eDNA of 3.610 to 2109 copies µL^−1^, with CVs of 0.53–11.05% among technical triplicates. All putative field detections were below the LOQ and were classified as detected but not quantifiable. Among 21 paired stations, putative target-DNA detection frequencies were 19.05% (4/21) in April and 47.62% (10/21) in September, but the difference was not significant (exact McNemar test, *p* = 0.070). A stock-enhancement release between surveys confounded the comparison. Thus, the observed difference cannot be confidently attributed to natural seasonal variation. Given that only one water sample was collected per station and each field extract was analyzed in a single dPCR reaction, these field observations should be considered preliminary and require further sampling and analytical replication.

## Simple Summary

The Chinese bahaba (*Bahaba taipingensis*) is one of the world’s most critically endangered marine fishes and is now extremely rare in the wild. Monitoring this species is challenging due to its extremely low density in turbid estuaries. Conventional capture-based survey methods have low detection probabilities and may harm this strictly protected species. Environmental DNA (eDNA) offers a non-invasive alternative, but detecting trace amounts of target DNA remains challenging. In this study, we developed a species-specific microfluidic digital PCR (dPCR) assay for detecting *B. taipingensis*. The assay was developed using muscle tissue samples and tested with water samples from three types of aquaculture systems before being preliminarily applied to field samples collected from natural estuarine habitats in the Pearl River Estuary (PRE). Putative target-DNA detections were observed at 4 of the 21 paired stations in April and 10 in September. However, this difference was not statistically significant in the paired analysis. All putative field detections were below the quantification limit, and signals consistent with target DNA were found at multiple stations. These field results should be considered preliminary because only one water sample was collected per station and each DNA extract was analyzed in a single dPCR reaction.

## 1. Introduction

The Chinese bahaba (*Bahaba taipingensis*), the largest sciaenid endemic to the coastal waters of China, was once distributed along the coasts of southeast China [1,2,3]. Over the past century, however, its population has declined severely because of historical overexploitation, predictable spawning aggregations that increased its vulnerability to fishing, the exceptionally high commercial value of its swim bladder, and widespread degradation of estuarine habitats [1,2,4,5]. Consequently, *B. taipingensis* is now rarely encountered in the wild, and the Pearl River Estuary (PRE) is regarded as one of its last known refuges [6,7]. The species is closely associated with estuarine ecosystems throughout its life cycle. Adults migrate inshore to form spawning aggregations during the breeding season, while juveniles use shallow estuarine habitats as nursery grounds before moving offshore [1,7]. Reflecting its severe decline, *B. taipingensis* is currently classified as Critically Endangered on the IUCN Red List [8] and is listed as a Class I nationally protected wildlife species [3,6].

Designing effective conservation strategies for *B. taipingensis* requires a clear understanding of its distribution, habitat use, and population trends [9]. Yet, monitoring this rare species in the highly dynamic and turbid waters of the Pearl River Estuary remains challenging. Traditional capture-based surveys generally have low detection probabilities for rare species and may be invasive or harmful, which is particularly problematic for a critically endangered and strictly protected fish [10,11]. Much of the current knowledge of *B. taipingensis* is derived from fishery-dependent records, which provide only a limited picture of its current distribution and population status [1]. Visual surveys are also limited by poor water clarity [12,13]. Although acoustic monitoring may help detect vocalizing adults during the spawning season, it provides limited information on juvenile habitats and movements outside the reproductive period [4,5,7,14]. As a result, the distribution and occurrence of *B. taipingensis* over space and time remain poorly understood, making it difficult to evaluate conservation measures such as marine protected areas and stock-enhancement programs.

Environmental DNA (eDNA) analysis is a non-invasive and sensitive tool for aquatic biomonitoring and can improve the detection of rare and cryptic species compared with conventional survey methods [11,15,16]. Previous studies in the Pearl River Estuary have demonstrated the feasibility of eDNA-based approaches for monitoring fish communities and characterizing spatial and temporal variation, while also highlighting the importance of sampling and DNA extraction procedures for detection performance [17,18,19]. However, applying eDNA in estuarine systems remains challenging. High concentrations of suspended particles, variable salinity, and potential PCR inhibitors can affect DNA persistence and amplification efficiency [20,21,22]. For a species as rare as *B. taipingensis*, eDNA is likely to occur at very low concentrations and may be particularly affected by dilution, transport, and degradation in dynamic estuarine environments [23,24].

Digital PCR (dPCR) has several features that are useful for detecting low-copy targets. By partitioning a sample into thousands of independent microreactions, dPCR allows absolute target quantification without an external standard curve. Compared with quantitative PCR (qPCR), dPCR can provide improved sensitivity and precision at low target concentrations and may be less affected by PCR inhibitors, depending on the assay and sample matrix [25,26,27,28]. These features make dPCR particularly useful for detecting low concentrations of eDNA in challenging samples such as turbid estuarine water.

To our knowledge, no species-specific dPCR assay has been reported for detecting *B. taipingensis* eDNA. This study aimed to develop a species-specific microfluidic dPCR assay targeting the mitochondrial *COI* region of *B. taipingensis* and to evaluate its analytical specificity and performance, including tests with tissue DNA from *B. taipingensis* and seven sympatric non-target sciaenid species. We then evaluated the assay using three aquaculture water matrices and preliminarily applied it to environmental water samples collected during two field surveys in the Pearl River Estuary (PRE). The field surveys provided a preliminary assessment of the assay’s ability to detect low levels of *B. taipingensis* eDNA under natural estuarine conditions. Given the potential for eDNA transport, the low target concentrations, and the stock-enhancement release between the two surveys, field detections were interpreted as evidence of putative target DNA in the collected water samples rather than direct evidence of fish presence or local abundance. Accordingly, the observed differences between the two surveys cannot be attributed solely to natural seasonal variation. This study provides a species-specific, non-invasive molecular approach for detecting *B. taipingensis* eDNA and a basis for more highly replicated monitoring in estuarine ecosystems.

## 2. Materials and Methods

**Figure 1:**
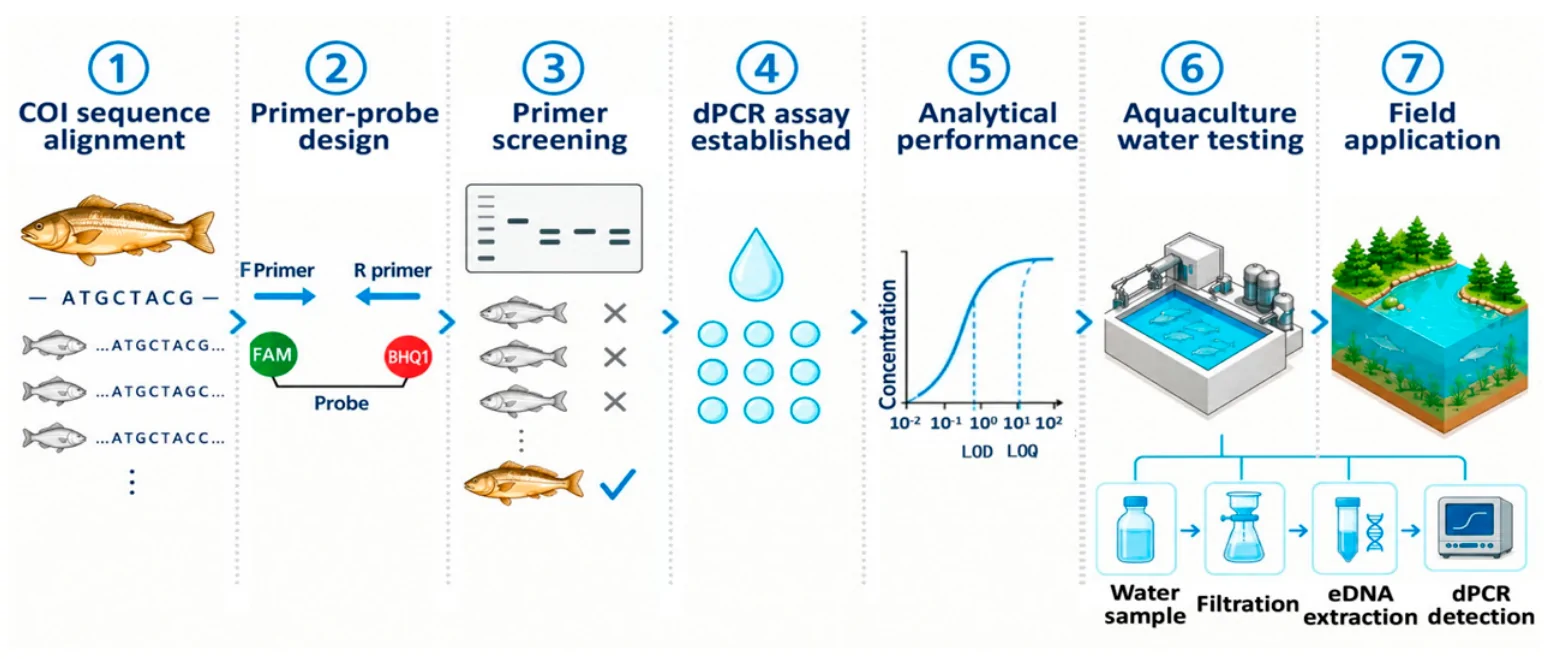
Overview of the workflow for the development, testing, and field application of the species-specific dPCR assay for *B. taipingensis*.

An overview of the study workflow, including assay development, analytical testing, and field application, is presented in Figure 1.

### 2.1. Sample Collection, Filtration, and DNA Extraction

DNA for assay development was extracted from muscle tissue of a single *B. taipingensis* specimen (total length, 52 cm; body weight, 1.019 kg) that was found dead following incidental capture during a legal local fishing operation. No *B. taipingensis* individuals were captured, handled, or sacrificed for this study, and the use of tissue from this incidentally obtained dead specimen did not require an additional specific collection permit. Tissue samples from seven sympatric sciaenid species were collected during the field campaigns and used for non-target specificity testing: goatee croaker (*Sciaena russelli*), spinyhead croaker (*Collichthys lucidus*), Caroun croaker (*Johnius carouna*), red drum (*Sciaenops ocellatus*), Belanger’s croaker (*Johnius belengerii*), yellow drum (*Nibea albiflora*), and amoy croaker (*Nibea miichthioides*). Tissue samples were preserved in 95% (*v*/*v*) ethanol and stored at −20 °C until DNA extraction. All specimens were initially identified based on external morphological characteristics. For non-target specimens that could not be reliably distinguished by morphology alone, taxonomic identity was further assessed by Sanger sequencing of the mitochondrial *COI* barcode region using the universal fish primers FishF1 and FishR1 [29]. Although its identity was already known, the *B. taipingensis* specimen was further confirmed using the same Sanger sequencing approach. The resulting sequences were queried against the NCBI GenBank database using BLAST (https://blast.ncbi.nlm.nih.gov/Blast.cgi, accessed on 25 September 2025), and species identity was assigned based on the closest reference matches. The sequences generated in this study were not deposited in GenBank and therefore do not have accession numbers. The closest GenBank reference matches, their accession numbers, and sequence identity values are provided in Table S1.

Water samples were collected at a depth of 1 m using a 5 L water sampler (Beijing Grasp Instrument and Equipment Co., Ltd., Beijing, China) and transported to the laboratory for filtration within 8 h. One field blank consisting of 1 L of ultrapure water was included in each of the two field surveys (April and September; *n* = 2 in total), transported alongside the environmental samples, and subsequently subjected to filtration, DNA extraction, and dPCR analysis to monitor potential contamination introduced during sample transport and laboratory processing.

**Figure 2:**
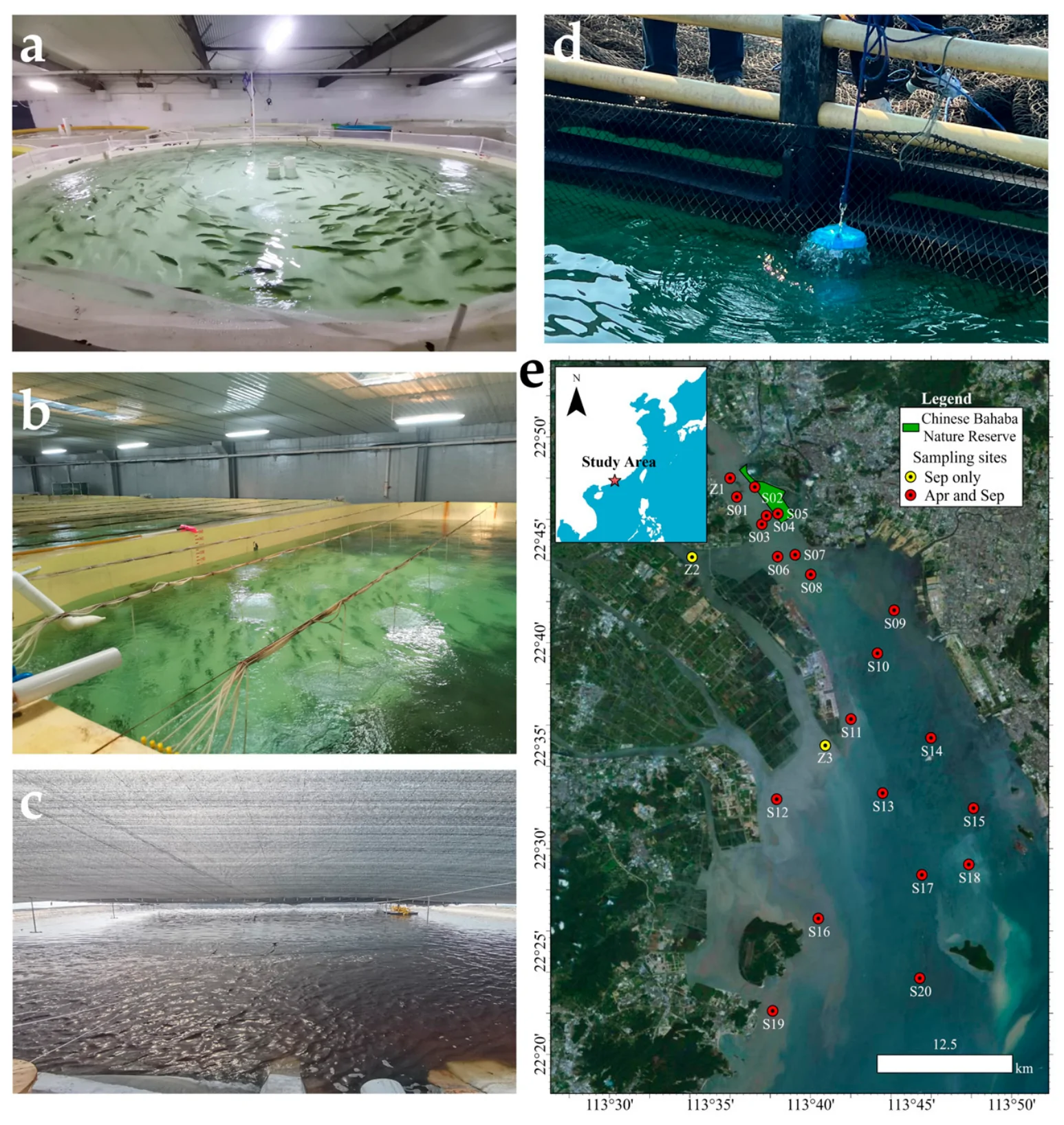
Aquaculture sampling environments and field sampling locations for *B. taipingensis*. (**a**–**d**) Three aquaculture systems: (**a**,**b**) recirculating aquaculture systems (RAS), (**c**) earthen ponds (EP), and (**d**) nearshore marine cage culture (NMCC); (**e**) spatial distribution of sampling stations in the Pearl River Estuary during the April and September 2025 field surveys. Yellow circles indicate stations sampled only in September, red circles indicate stations sampled in both April and September, and the green area denotes the Chinese bahaba Nature Reserve. The red star indicates the general location of the study area.

To test the applicability of the assay across contrasting water matrices, water samples were collected from three aquaculture systems rearing *B. taipingensis*: (i) indoor recirculating aquaculture systems (RAS; *n* = 8 sampling points, F1-F8, 1 L per point), representing a controlled, low-turbidity environment with relatively stable water quality (Figure 2a,b); (ii) outdoor earthen ponds (EP; *n* = 2 sampling points, T1 and T2, 0.5 L per point), representing relatively turbid pond-water conditions (Figure 2c); (iii) nearshore marine cage-culture sites (NMCC; *n* = 2 sampling points, H1 and H2, 1 L per point), representing an open coastal environment subject to seawater exchange and dilution (Figure 2d). These systems provided contrasting water matrices for testing assay applicability under different environmental conditions before field application.

Seawater samples were collected in April and September 2025 from the potential *B. taipingensis* habitat in the PRE. The April survey was conducted on 15–16 April 2025, during the reported spawning period, and included 21 locations from Humen to Nei Lingding Island. The same 21 stations were resampled on 5–6 September 2025, during the reported post-spawning period. Two additional stations were sampled only in September based on recent bycatch records and a documented stock-enhancement release event, resulting in a total of 23 stations in the September survey (Figure 2e). These two additional stations were not part of the 21-station paired survey design. At each station, a single 1 L seawater sample was collected for eDNA analysis.

Filter membrane types were selected according to water turbidity because membrane type can affect eDNA capture and filtration performance [15,30]. For low-turbidity RAS water, cellulose acetate (CA) membrane filters (0.45 μm, 47 mm; Shanghai Xingya Jinjing Instrument Co., Ltd., Shanghai, China) were used. For outdoor earthen ponds, nearshore marine cage-culture sites, and natural habitat samples, mixed cellulose ester (MCE) membranes (0.45 μm, 47 mm; Cytiva, Marlborough, MA, USA) were used because of their higher particle-loading capacity in turbid water eDNA studies [31,32]. To minimize cross-contamination risk, the filtration apparatus was decontaminated between samples with 5% sodium hypochlorite and thoroughly rinsed with ultrapure water. Before filtration, the apparatus was additionally pre-rinsed with a small volume of the water sample to be filtered to minimize dilution by residual ultrapure water. Following filtration, membranes were folded with the particle-retaining side inward, transferred to sterile 2 mL cryovials, and immediately frozen in liquid nitrogen until DNA extraction, which was completed within two weeks.

DNA was extracted from filter membranes using the TIANamp Marine Animals DNA Kit (Tiangen Biotech, Beijing, China) with modifications to the manufacturer’s protocol. Each membrane was cut into small pieces with sterile scissors and digested in 800 µL GA buffer and 80 µL Proteinase K at 56 °C with shaking at 1200 rpm for 2.5 h. After purification, DNA was eluted in 100 µL TE buffer, and the eluate was reapplied to the same silica column for a second elution, yielding a final eluate volume of 100 µL. DNA from muscle tissue was extracted following the manufacturer’s standard protocol and eluted in 200 µL TE buffer. The concentration and purity of all DNA samples were assessed using a NanoDrop spectrophotometer (Thermo Fisher Scientific, Waltham, MA, USA). The two field blanks described above were filtered and then processed using the same DNA extraction and dPCR procedures as the environmental samples. No separate extraction-only blanks were included.

### 2.2. Primer Screening, Probe Design, and Specificity Testing

Complete mitochondrial genome sequences of *B. taipingensis* and the seven sympatric sciaenid species described above were retrieved from the NCBI GenBank database and aligned using MEGA 11. The mitochondrial *COI* gene, a widely used DNA barcode for animals [33], was examined to identify nucleotide differences specific to *B. taipingensis* in regions suitable for primer design. Based on the sequence alignment, nine candidate primer pairs were designed using Primer Premier 5.0 [34]. Candidate primers were selected considering predicted melting temperature (Tm; 50–60 °C), GC content (40–60%), amplicon length (100–250 bp), and the presence of species-discriminating nucleotide sites. Their thermodynamic properties and potential secondary structures, including hairpins, self-dimers, and heterodimers, were further evaluated using Oligo 6.0 [35]. Candidate pairs were designed to have similar predicted Tm values and were subsequently screened at a common annealing temperature of 55 °C (Table S2). In addition, all nine candidate primer pairs were subjected to in silico specificity assessment using NCBI Primer-BLAST (https://www.ncbi.nlm.nih.gov/tools/primer-blast/index.cgi?LINK_LOC=BlastHome, accessed on 27 September 2025) under the default specificity-checking parameters against publicly available nucleotide sequences, with particular attention to *B. taipingensis*, closely related sciaenid species, and other potential non-target organisms. No significant predicted amplification of non-target sequences was identified under the Primer-BLAST search conditions.

No experimental temperature-gradient optimization was performed, and all nine candidate primer pairs were screened by conventional PCR at an annealing temperature of 55 °C using genomic DNA from *B. taipingensis* and seven non-target sciaenid species. The closest relative, *N. miichthioides*, was tested in triplicate to further assess specificity. PCR reactions were performed in a 25 µL volume containing 12.5 µL of 2× ProTaq Master Mix (Accurate Biology, Changsha, China), 1.0 µL of each primer (10 µM), 50 ng of template DNA, and nuclease-free water to the final volume. Two no-template controls were included in each run: one prepared in a dedicated PCR workstation and one exposed to laboratory air for approximately 5 s to monitor aerosol contamination. Cycling conditions were 94 °C for 1 min; 35 cycles of 98 °C for 10 s, 55 °C for 30 s, and 72 °C for 30 s; and a final extension at 72 °C for 2 min. Amplicons were visualized on 1.5% agarose gels stained with 1× GeneGreen (Tiangen Biotech, China). Following primer screening, the amplicon generated from *B. taipingensis* genomic DNA by the final selected primer pair was purified and subjected to Sanger sequencing. The resulting sequence was queried against the NCBI GenBank database using BLAST to confirm that the selected primer pair amplified the intended *B. taipingensis COI* target region. Following selection of the primer pair, a species-specific TaqMan probe was designed within the target amplicon, targeting a region containing nucleotide sites that discriminate *B. taipingensis* from the non-target species examined. The probe was labeled with 6-carboxyfluorescein (FAM) at the 5′ end and Black Hole Quencher 1 (BHQ1) at the 3′ end and was selected based on appropriate melting temperature and minimal secondary structure formation.

### 2.3. dPCR Assay Development

Development, analytical evaluation, and reporting of the dPCR assay were guided by the digital MIQE Recommendations [36]. Primer and probe concentrations were selected based on the manufacturer’s recommendations and previously published dPCR assays [37,38,39]. For the dPCR assay, an annealing temperature of 55 °C was selected based on in silico thermodynamic analyses. No experimental temperature-gradient optimization of the dPCR annealing temperature was performed. The predicted dPCR primer melting temperature (Tm) of the primer pair was 56 °C (Thermo Fisher Scientific Tm Calculator, https://www.thermofisher.com/us/en/home/brands/thermo-scientific/molecular-biology/molecular-biology-learning-center/molecular-biology-resource-library/thermo-scientific-web-tools/tm-calculator.html, accessed on 1 January 2026). Potential interactions between the forward and reverse primers were also assessed using DINAMelt at 55 °C with 50 mM Na^+^ and 3 mM Mg^2+,^ which predicted no significant hybridization between the two primer sequences.

The dPCR assay was performed using an SG-2000 microfluidic digital PCR System (RainSure Scientific Biotechnology Co., Ltd., Suzhou, China). For assay development and analytical performance testing, each 20 µL reaction contained 5 µL of 4× dPCR Master Mix (RainSure Scientific), 1.8 µL each of forward and reverse primers (10 µM stock; final concentration 0.9 µM each), 0.5 µL of probe (10 µM stock; final concentration 0.25 µM), 3 µL of template DNA, and nuclease-free water to the final volume. According to the manufacturer’s specifications, each reaction was nominally partitioned into approximately 20,000 partitions, each with a nominal volume of 1 nL. The nominal partition volume was used by GeneCountSoft v1.66.0828 (RainSure Scientific, Suzhou, China) for Poisson-based concentration estimation, whereas the actual number of accepted partitions was determined separately for each reaction after quality control filtering. Templates included *B. taipingensis* tissue DNA as the target control, DNA from the seven sympatric sciaenid species as non-target controls, and a no-template control (NTC) to monitor contamination. The reaction mixture was loaded onto a dPCR chip according to the manufacturer’s instructions. Amplification was performed on the SG-2000 system under the following conditions: 95 °C for 10 min; 45 cycles of 94 °C for 30 s and 55 °C for 1 min; followed by 98 °C for 10 min and 20 °C for 2 min. Endpoint fluorescence signals were acquired using a Dscanner4–1000 biochip reader and analyzed with GeneCountSoft v1.66.0828 (RainSure Scientific, China).

### 2.4. Analytical Performance of the dPCR Assay

For assessment of assay linearity, the dPCR assay was evaluated using serially diluted *B. taipingensis* genomic DNA under the reaction conditions described in Section 2.3. A seven-level ten-fold serial dilution series (10^−1^–10^−7^) was prepared from the 90 ng µL^−1^ genomic DNA stock. Linearity was assessed by regression analysis of log_10_-transformed reaction concentrations (copies µL^−1^ per reaction) against log_10_-transformed dilution levels. The coefficient of determination (*R*^2^), regression slope, 95% confidence interval (CI), and significance of the regression model were calculated. The range evaluated for assay linearity was defined by the concentrations with detectable target-positive partitions in the seven-level serial dilution experiment, including measured concentrations below the independently established LOQ. According to the manufacturer’s specifications, the theoretical upper measurement limit was approximately 5000 copies µL^−1^ per reaction, but this theoretical limit was not independently validated in the present study.

Additional low-concentration dilution levels (10^−4^, 4 × 10^−5^, 2 × 10^−5^ and 10^−5^) were evaluated to determine the limit of detection (LOD) and limit of quantification (LOQ), with eight technical replicate reactions analyzed at each level. The blank dataset comprised 20 NTC reactions collected across multiple independent dPCR runs and 10 dPCR reactions performed on two independent field blank DNA extracts (five replicate reactions per extract; one field blank from each survey). The limit of blank (LOB) was operationally defined as the highest apparent target concentration observed among all blank reactions. A dPCR reaction was considered detected when at least one accepted target-positive partition was identified above the analytical fluorescence threshold. The LOD criterion was predefined as ≥95% replicate positivity [36,40]. Because only eight replicate reactions were tested at each concentration, this criterion was met only when all eight replicates were positive (8/8, 100%). Exact binomial (Clopper-Pearson) 95% confidence intervals were calculated for the observed detection proportion at each low-concentration level. The LOD was reported as the lowest tested concentration meeting this predefined criterion under the experimental conditions, rather than as a precise estimate of the concentration corresponding to a 95% detection probability. The LOQ was defined as the lowest tested concentration that met the LOD criterion and had a coefficient of variation (CV) ≤ 35% across all eight technical replicates [40]. Reactions with no accepted target-positive partitions were assigned a measured concentration of zero and were retained when calculating the mean concentration, standard deviation, and CV.

### 2.5. Assay Application in Aquaculture Water Samples

To test the assay with different aquaculture water matrices, DNA extracts from water samples collected from the three aquaculture systems (RAS, EP, and NMCC) were analyzed directly without additional purification or inhibitor-removal steps. No independent inhibition assessment or spike-and-recovery assessment was performed. Therefore, these experiments were not considered a validation of matrix-specific recovery or inhibition. Each sample was analyzed in triplicate using the established dPCR workflow. For each technical replicate, 10.9 µL of DNA extract was used as the template input, except for four RAS samples (F5, F6, F7 and F8), which yielded target concentrations exceeding the manufacturer-specified theoretical upper measurement limit of 5000 copies µL^−1^ in preliminary testing, and their template input volume was therefore reduced to 3 µL. In all cases, the total reaction volume was kept constant at 20 µL, with the difference in template volume compensated by adding the corresponding volume of nuclease-free water, and all other reaction components remained unchanged. eDNA concentrations in the original water samples were calculated using the actual template input volume for each sample (3 µL or 10.9 µL), as described in Section 2.7.

### 2.6. Application of the dPCR Assay to Field eDNA Samples

The established dPCR assay was preliminarily applied to field eDNA samples collected from the potential habitat of *B. taipingensis* during the April and September 2025 surveys. For all field samples, 10.9 µL of DNA extract was added per reaction to increase the amount of environmental DNA represented in each dPCR analysis. Each field DNA extract was analyzed in a single dPCR reaction. Each run included a no-template control (NTC) and a positive control containing genomic DNA extracted from *B. taipingensis* muscle tissue. The field blank associated with each survey was analyzed separately in five dPCR reactions as a process control. A field reaction was operationally classified as detected when at least one accepted target-positive partition was identified above the analytical fluorescence threshold, according to the detection and data-processing criteria described in Section 2.4 and Section 2.7. A field result was considered valid only when the corresponding NTC contained no target-positive partitions, the positive control produced the expected target-positive signal, and all five dPCR reactions from the field blank associated with that survey contained no target-positive partitions.

### 2.7. Data Processing and Statistical Analysis

Partition quality was evaluated according to the predefined quality-control criteria of the instrument manufacturer. Only reactions in which the measured droplet diameters fell within the accepted range of 80–110 µm and the droplet-size coefficient of variation was ≤3% were included in the analysis. The total number of accepted partitions was recorded for each reaction automatically. Raw endpoint fluorescence data were exported from GeneCountSoft v1.66.0828 (RainSure Scientific, China) and partition classification was performed using the software’s default automated thresholding algorithm. The automatically assigned fluorescence threshold was retained for all reactions, and no manual threshold adjustments were made. Fluorescence-amplitude plots were subsequently inspected only as a quality-control step to identify obvious technical artifacts, such as signals associated with dust or bubbles. A reaction was classified as target-positive when at least one accepted partition was classified as positive by the predefined analytical procedure, provided that the corresponding NTC and non-target control showed no target-positive partitions and the positive control produced the expected signal. For field samples, the associated field blank was additionally required to show no target-positive partitions.

Reaction-level concentrations (*C_rxn_*, copies μL^−1^ per reaction), were calculated automatically by GeneCountSoft based on Poisson statistics. Environmental concentrations in the original water samples (*C_env_*, copies L^−1^) were calculated as *C_env_* = *C_rxn_* × (*V_rxn_*/*V_template_*) × (*V_elution_*/*V_filtered_*), where *V_rxn_* is the total reaction volume (20 µL), *V_template_* is the template input volume (3 µL or 10.9 µL), *V_elution_* is the final DNA elution volume (µL), and *V_filtered_* is the filtered water volume (L). Based on the analytical performance criteria, samples were classified as quantifiable (*C_rxn_* ≥ LOQ), detected but not quantifiable (DNQ; LOB < *C_rxn_* < LOQ), or not detected (ND; *C_rxn_* ≤ LOB).

For aquaculture samples, which were analyzed in triplicate, the mean, standard deviation (SD), and coefficient of variation (CV) of *C_rxn_* were calculated for each sample. Technical repeatability was examined by plotting replicate measurements and mean ± SD on a log_10_ scale. The relationship between CV and mean *C_rxn_* was described using the empirical power-law model CV = *a C ^b^*, where C denotes the mean *C_rxn_*, *a* is the fitted scaling coefficient, and *b* is the exponent describing the dependence of CV on concentration. The model was fitted by ordinary least-squares regression after log_10_ transformation of both CV and *C_rxn_*. CV values of 5% and 10% were included as descriptive reference levels for visualization of technical precision, these values were not used as predefined assay acceptance criteria. Similarly, the relationship between SD and mean *C_rxn_* was described using the power-law model SD = *k C ^n^* where *k* is the fitted scaling coefficient and *n* is the exponent describing how SD changes with concentration. This model was fitted by ordinary least-squares linear regression after log_10_ transformation of SD and *C_rxn_*. The coefficient of determination (*R*^2^) was calculated on the transformed scale. The estimated slope *n* was compared descriptively with theoretical reference values of 0.5, corresponding to Poisson counting behavior, and 1.0, representing constant relative variation. These analyses were used to characterize technical repeatability only and were not considered evidence of matrix-specific recovery, absence of inhibition, or full validation across the three aquaculture matrices. No inferential comparison among aquaculture systems was performed. All analyses were performed in Python 3.12 (NumPy, pandas, SciPy, Matplotlib).

For the field survey, statistical analysis was restricted to detection/non-detection status because all field detections were below LOQ and each field DNA extract was analyzed in a single dPCR reaction. For statistical analysis, DNQ reactions were treated as detections and ND reactions as non-detections. The primary comparison between the April and September 2025 surveys was based on the 21 stations sampled on both occasions. Changes in paired detection status were evaluated using a two-sided exact McNemar test. The two stations sampled only in September (Z2 and Z3) were excluded from the paired analysis. Detection frequencies were calculated as the proportion of stations classified as detected, and 95% confidence intervals for these proportions were calculated using the Wilson score method. Paired changes in detection status between surveys were additionally visualized using an alluvial diagram, with ribbon widths proportional to the number of stations in each transition category. A *p* value < 0.05 was considered statistically significant.

## 3. Results

### 3.1. Species-Specific Primer–Probe Selection

**Figure 3:**
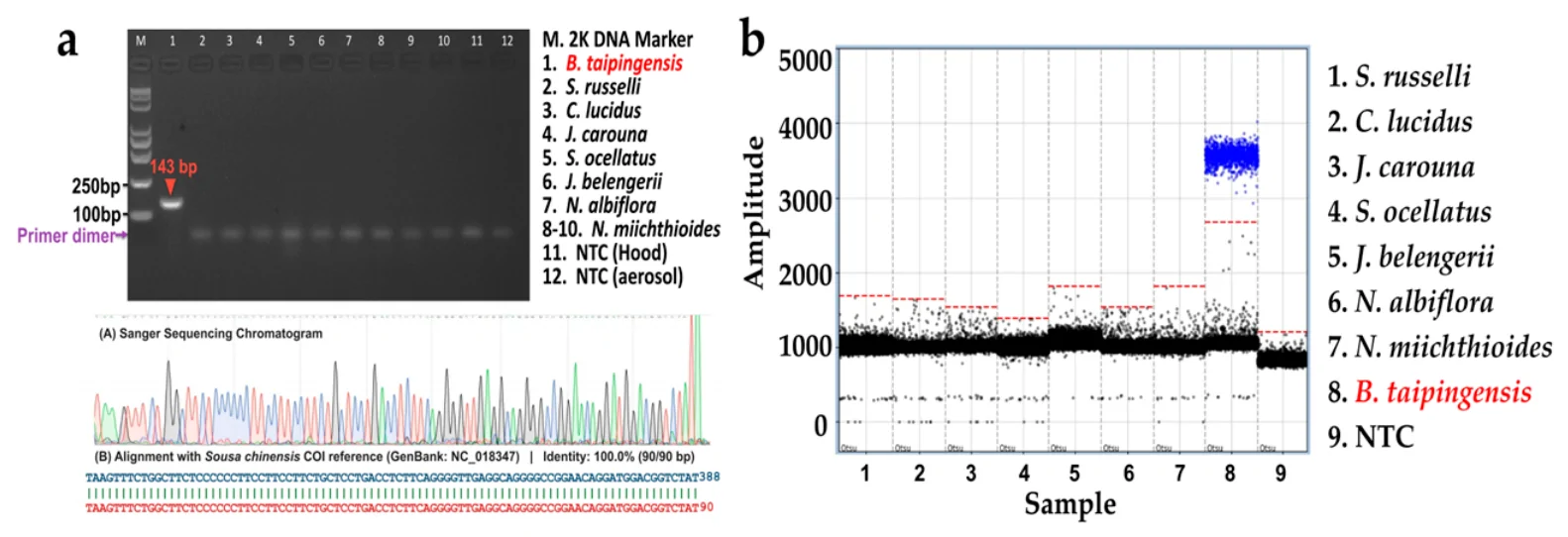
Primer selection and specificity testing of the dPCR assay for *B. taipingensis*. (**a**) Primer screening and Sanger sequence of the selected 143 bp *COI* amplicon, showing alignment with the *B. taipingensis COI* reference sequence (GenBank: NC_018347). The red arrow indicates the target 143 bp *COI* amplicon of *B. taipingensis*; (**b**) dPCR specificity testing using genomic DNA from *B. taipingensis*, seven non-target sciaenid species, and a no-template control (NTC) in the same dedicated PCR workstation. The red dashed line represents the threshold for positive partition discrimination, blue dots indicate positive partitions, and black dots indicate negative partitions.

Among the nine candidate primer pairs targeting the mitochondrial *COI* gene (Table S2), only one primer pair (F6: 5′-TTGTGCCCCTAATAATTGGT-3′; R6: 5′-ATAGACCGTCCATCCTGTTC-3′) generated the expected 143 bp amplicon from *B. taipingensis* genomic DNA, with no corresponding amplicon detected from the seven sympatric non-target sciaenid species tested, including the closely related *N. miichthioides*, or in the NTCs (Figure 3a and Figure S1). The 143 bp amplicon was confirmed by Sanger sequencing and showed 100% sequence identity to the reference mitochondrial *COI* sequence of *B. taipingensis* (GenBank accession NC_018347).

The selected primer pair was subsequently combined with a species-specific hydrolysis probe (5′-FAM-CTGACATGGCATTCCCCCGAATAA-BHQ1-3′) for dPCR assay development. In the probe-based dPCR assay, *B. taipingensis* genomic DNA produced a distinct positive-partition cluster that was clearly separated from the negative cluster (Figure 3b). No positive partitions were observed for any of the seven tested non-target sciaenid species or for the NTC. These results supported the specificity against the seven non-target species tested and showed clear endpoint fluorescence separation under the tested dPCR conditions.

### 3.2. Analytical Performance, Linearity, and Detection Limits of the dPCR Assay

**Figure 4:**
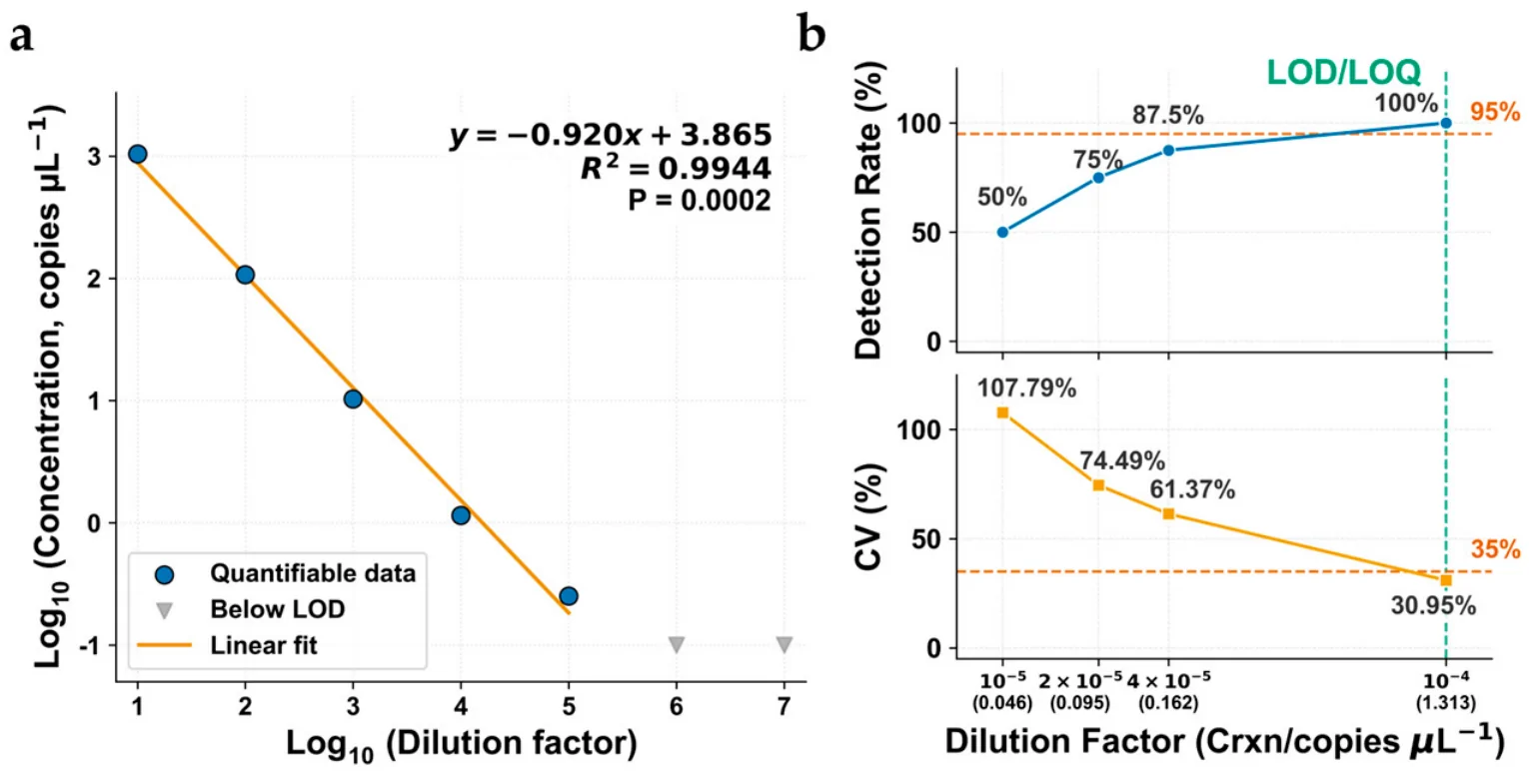
Analytical characterization of the species-specific dPCR assay for *B. taipingensis*. (**a**) Relationship between measured dPCR concentration and dilution level across the tested serial dilution series. The fitted regression equation, coefficient of determination (*R*^2^), and *p* value are shown; (**b**) detection rate and coefficient of variation (CV) across the tested low-concentration levels. Dashed horizontal lines indicate the predefined ≥95% replicate-detection criterion for LOD assessment and the ≤35% CV criterion for LOQ assessment.

In the seven-level serial dilution experiment, target-positive partitions were detected at five dilution levels (10^−1^ to 10^−5^), whereas no positive partitions were observed at 10^−6^ or 10^−7^. Across the detectable dilution range (10^−1^ to 10^−5^), measured reaction-level concentrations decreased from 1043 to 0.252 copies µL^−1^. Log_10_-transformed measured concentration showed a strong linear relationship with log_10_-transformed dilution level (*R*^2^ = 0.9944; slope = −0.920 ± 0.040; 95% CI, −1.047 to −0.793; *p* < 0.001; Figure 4a). The 95% confidence interval of the fitted slope included the theoretical value of −1, indicating no statistically supported deviation from proportional ten-fold dilution over the evaluated range.

No target-positive partitions were detected in any of the 30 blank controls, comprising 20 NTC reactions and 10 dPCR reactions from two independent field blank DNA extracts (one blank from each field survey and five replicate dPCR reactions per extract) (Table S3). LOB was therefore zero under the definition used in this study. At the low-concentration levels used for LOD and LOQ assessment, all eight replicate reactions were positive at a mean *C_rxn_* of 1.313 copies µL^−1^ (8/8; detection rate, 100%; exact 95% CI, 63.06–100.0%; Table A1), and the CV among replicates was 30.95% (Figure 4b; Table A2). This was the lowest tested concentration satisfying both the operational LOD criterion (≥95% observed replicate positivity) and the LOQ precision criterion (CV ≤ 35%). At the next lower tested concentration, 0.162 copies µL^−1^, seven of eight replicates were positive (7/8; detection rate, 87.5%; exact 95% CI, 47.35–99.72%), which did not meet this criterion for LOQ. Since no additional concentrations were tested between these two levels, the LOD was bounded as 0.162 < LOD ≤ 1.313 copies µL^−1^ and the LOQ was 1.313 copies µL^−1^. Raw reaction-level concentration data for the four low-concentration dilution levels are provided in Table A1 and Table A2, while complete analytical details, including reaction status, channel, measured concentration, positive and negative partition counts, accepted partition counts, and fluorescence thresholds, are provided in Table S3.

### 3.3. Application of the dPCR Assay to eDNA Detection in Aquaculture Water Samples

**Figure 5:**
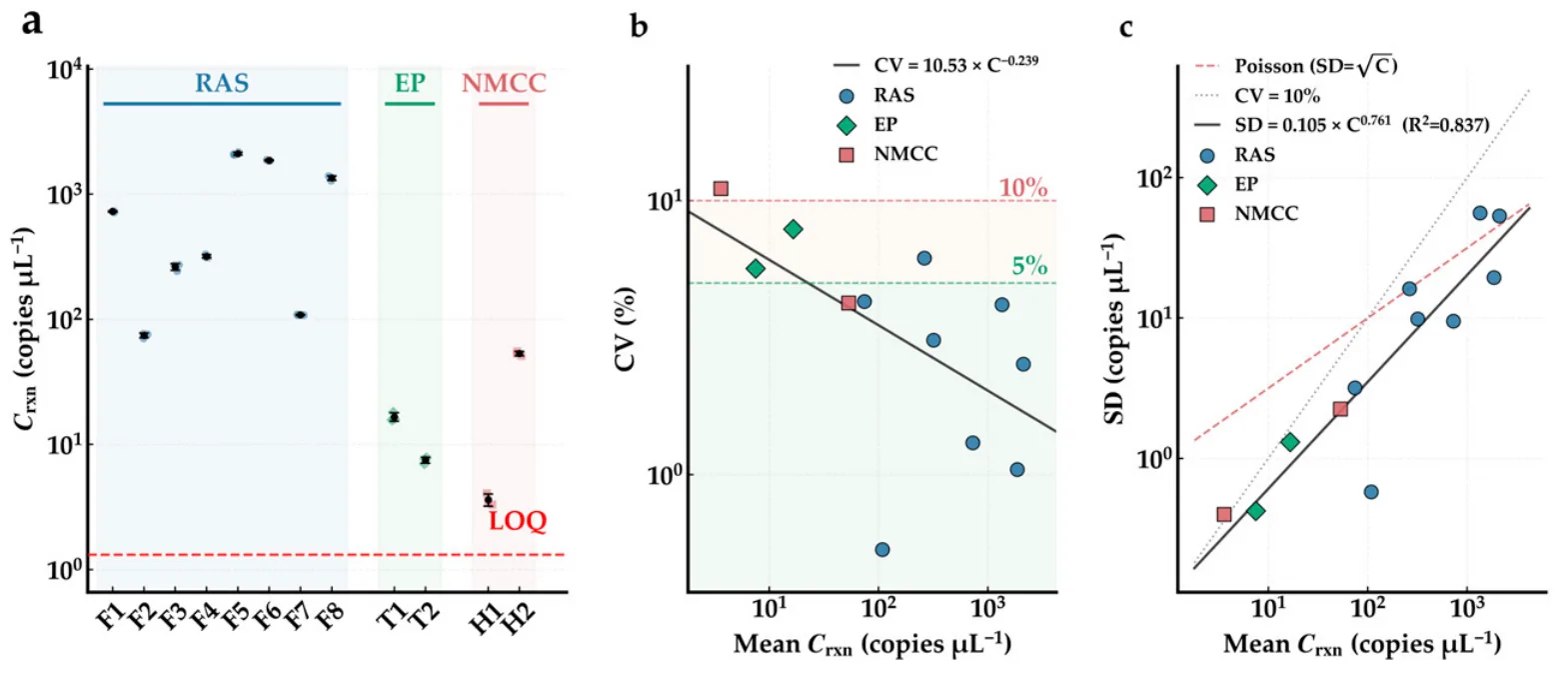
Application of the dPCR assay for *B. taipingensis* eDNA detection in three aquaculture water samples. (**a**) Reaction-level concentrations (*C_rxn_*, copies µL^−1^) measured in 12 water samples collected from three aquaculture systems. Colored markers represent individual technical replicate measurements (*n* = 3 per sample), and black dots with vertical bars indicate the mean ± standard deviation (SD). The horizontal red dashed line indicates the experimentally established limit of quantification (LOQ); (**b**) relationship between the coefficient of variation (CV) among triplicate reactions and the corresponding mean reaction-level concentration (*C_rxn_*) on a log–log scale. The solid line represents the fitted power-law relationship (CV = 10.53 × *C*^−0.239^) obtained by ordinary least-squares regression of log_10_-transformed CV values against log_10_-transformed *C_rxn_* values. Horizontal dashed lines at CV = 5% (green) and CV = 10% (red) are shown as descriptive reference levels and were not used as predefined assay acceptance criteria; (**c**) relationship between the SD and mean *C_rxn_* of triplicate measurements on a log–log scale. The solid black line shows the fitted power-law model (SD = 0.105 × *C*^0.761^, *R^2^* = 0.837), obtained by ordinary least-squares regression of log_10_-transformed SD values against log_10_-transformed *C_rxn_* values. Reference lines with slopes of 0.5 (red dashed line) and 1.0 (gray dotted line) are shown for descriptive comparison with Poisson counting behavior and constant relative variability, respectively. In all panels, marker shapes and colors indicate the three aquaculture systems as defined in panel (**a**).

The dPCR assay detected *B. taipingensis* eDNA in all 12 water samples collected from the three aquaculture systems and all samples were classified as quantifiable (Figure 5a; Table A3). Mean reaction-level concentrations (*C_rxn_*) ranged from 3.610 to 2109 copies µL^−1^, with the lowest concentration detected at site H1 and the highest at site F5. After conversion to concentrations in the original water samples using the corresponding template input, DNA elution volume, and filtered water volume, environmental concentration (*C_env_*) ranged from 6.62 × 10^2^ to 1.41 × 10^6^ copies L^−1^, spanning more than three orders of magnitude across the three aquaculture systems.

Among the technical triplicates, CVs ranged from 0.53% to 11.05%, with a mean of 4.328%. Eleven of the 12 samples (91.7%) had CVs below 10% and eight of 12 samples (66.7%) had CVs below 5%. The relationship between CV and mean *C_rxn_* was CV = 10.53 × *C*^−0.239^ (Figure 5b), showing a decrease in relative variability with increasing measured concentration over the observed concentration range. The relationship between the SD of triplicate measurements and mean *C_rxn_* was described by SD = 0.105 × *C*^0.761^ (*R*^2^ = 0.837; slope 95% CI: 0.536–0.986; Figure 5c). The estimated exponent of 0.761 fell between the descriptive reference values of 0.5 for Poisson counting behavior and 1.0 for constant relative variability. These analyses describe the technical repeatability observed among triplicate reactions for the 12 aquaculture samples and were not used as tests of differences among aquaculture systems. No independent inhibition or spike-and-recovery assessment was performed. Thus, the aquaculture experiment demonstrates assay application and technical repeatability under the tested conditions but does not provide matrix-specific estimates of DNA recovery or PCR inhibition.

### 3.4. Differences in B. taipingensis eDNA Between the April and September Surveys

**Figure 6:**
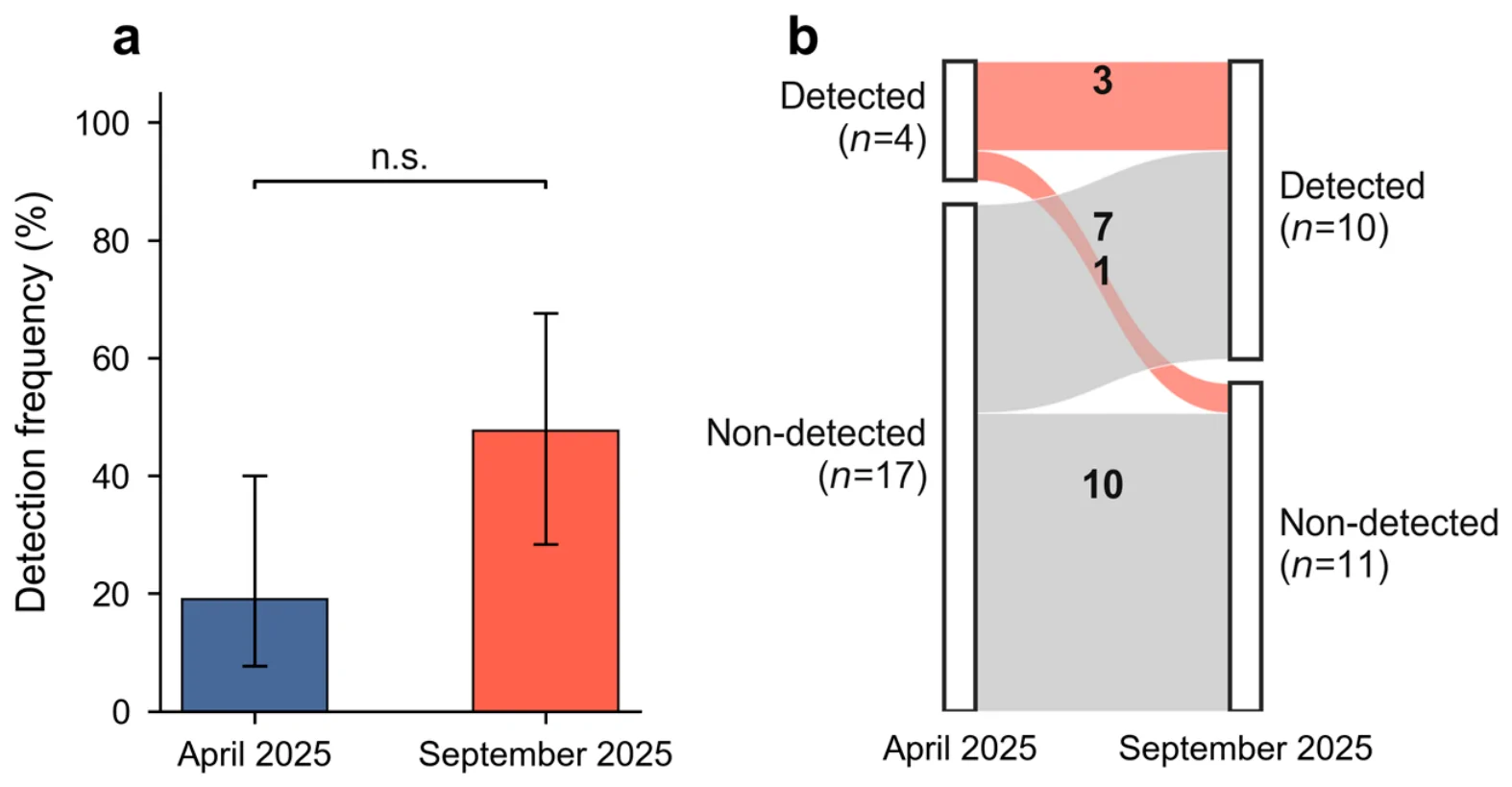
Differences in *B. taipingensis* eDNA detection between the April and September 2025 surveys in the Pearl River Estuary. (**a**) Detection frequency among the 21 paired stations in April 2025 and September 2025. Bars represent the proportion of stations classified as detected, and error bars represent 95% Wilson confidence intervals. Stations Z2 and Z3, sampled only in September, were excluded from the paired comparison. Differences between the two surveys were evaluated using a two-sided exact McNemar test (*p* = 0.070, n.s.). (**b**) Alluvial diagram showing detection-status transitions between April and September based on the same 21 paired stations. Ribbon widths are proportional to the number of stations in each transition category, and numbers within ribbons indicate station counts.

Putative target-DNA signals were observed during both field surveys. All positive field detections were below the experimentally established LOQ and were therefore classified as DNQ, whereas samples without target-positive signals were classified as ND (Table 1). Consequently, comparisons between surveys were based on binary detection status rather than quantitative eDNA concentrations. Among the 21 stations sampled in both surveys, putative target-DNA detections were observed at four stations (19.05%) in April and ten stations (47.62%) in September (Figure 6a). Three stations showed putative target-DNA detections in both surveys, one changed from a putative detection in April to ND in September, seven changed from ND in April to a putative detection in September, and ten were ND in both surveys (Figure 6b). Although detections were more frequent in September, the difference in paired detection status was not statistically significant according to the two-sided exact McNemar test (*p* = 0.070).

Two additional stations, Z2 and Z3, were sampled only in September and were therefore excluded from the paired statistical analysis. Both additional stations were classified as DNQ, resulting in detections at 12 of the 23 stations (52.2%) sampled in September when all September stations were considered descriptively. Because these stations were not included in the April survey, the 12/23 value was not used for statistical comparison between surveys.

## 4. Discussion

This study developed a species-specific dPCR assay for *B. taipingensis*, evaluated its analytical performance, and assessed its application in aquaculture water samples and natural estuarine water samples. The assay showed no cross-amplification with the seven non-target sciaenid species tested and good technical repeatability among triplicate reactions from aquaculture water samples. In the field surveys, putative target-DNA signals were observed in samples from both April and September. However, all field detections were below the experimentally established LOQ, and each station was represented by a single 1 L water sample analyzed in a single dPCR reaction. The field results should therefore be considered preliminary evidence of target DNA in the collected field water samples rather than estimates of eDNA concentration, local abundance, or confirmed presence of living *B. taipingensis* at individual sampling stations. Low-copy eDNA detections may nevertheless contribute useful information to rare-species monitoring when interpreted together with analytical controls, repeated sampling, and independent ecological evidence [30,41,42,43,44]. This distinction is particularly important for critically endangered species occurring at very low densities, for which target DNA may frequently occur near the analytical detection limit.

Interpretation of the DNQ field signals requires particular caution because stochastic amplification, sampling heterogeneity, low-level contamination, and other sources of uncertainty become increasingly important at very low target concentrations [30,40,45]. In the present study, no target-positive partitions were observed in the NTCs or field blanks analyzed, providing no evidence of contamination in these controls. Putative target DNA signals were observed at three stations in both surveys, suggesting recurrence of the signal across the two sampling occasions. However, repeated detection across two sampling occasions does not exclude stochastic detection or other sources of uncertainty, nor does it validate the remaining detections that occurred during only one survey. Moreover, the repeated observations were obtained months apart rather than from independent water replicates collected during the same sampling event. Given that each field station was represented by only one water sample and each extract by only one dPCR reaction, the present data do not permit direct estimation of within-site sampling variability or analytical repeatability. Independent water replicates and multiple dPCR reactions per extract would provide stronger support for future low-copy field detections [40,42,45,46].

Putative target-DNA detections were more frequent among the 21 paired stations in September (10/21) than in April (4/21), but the difference in paired detection status was not statistically significant (two-sided exact McNemar test, *p* = 0.070). The observed difference between the two surveys therefore does not provide sufficient evidence to conclude that natural seasonal variation was responsible. Interpretation is further complicated by the stock-enhancement release that occurred between the April and September surveys. September detections could potentially originate from released individuals, movement or mortality of released fish, or DNA introduced through transport and release operations [47,48]. The effects of stock enhancement also cannot be separated from changes in environmental conditions, eDNA shedding, degradation, tidal transport, freshwater discharge, or sampling variability between surveys [47,48,49,50,51,52,53]. The two additional stations sampled only in September, Z2 and Z3, both yielded DNQ signals, but these stations were selected based on recent bycatch information and the stock-enhancement event and therefore did not follow the same sampling framework as the 21 paired stations. Their detections were treated descriptively and excluded from the paired statistical comparison. Because these signals were also below the LOQ and obtained after the release event, they cannot be attributed specifically to wild individuals. Repeated surveys across multiple time points and years, preferably during periods unaffected by recent stock enhancement, will be needed to determine whether reproducible temporal patterns in *B. taipingensis* eDNA detection occur.

Hydrodynamic transport further limits the spatial interpretation of *B. taipingensis* eDNA in the Pearl River Estuary. In estuarine systems, extracellular DNA and DNA-containing biological material can be transported, diluted, resuspended, and degraded under the combined influence of tidal exchange, freshwater discharge, suspended sediments, and local circulation [22,50,51,52]. These processes may be especially important when target eDNA concentrations are very low. Detection of target DNA in a water sample therefore does not demonstrate that a living individual was present at the exact sampling location at the time of collection. Individual sampling stations should also not be interpreted as local abundance hotspots based on the present data. This limitation also applies to stations outside the immediate stock-enhancement area because released fish and their associated eDNA may move or be transported away from the release location. The assay is designed to detect *B. taipingensis* DNA and showed no cross-amplification among the seven non-target sciaenids tested, but it cannot distinguish DNA from wild and released individuals. Combining replicated eDNA sampling with hydrodynamic modelling and independent monitoring could improve identification of the likely sources and spatial distribution of detected DNA [51,54].

The aquaculture samples showed that the assay could detect and quantify *B. taipingensis* eDNA under the tested conditions in three contrasting culture systems, and technical triplicates generally showed low within-sample variation. However, these results do not represent validation of matrix-specific analytical performance. No inhibition assay, internal amplification control, dilution-based inhibition assessment, or spike-and-recovery experiment was conducted. PCR inhibition and DNA recovery efficiency were therefore not independently quantified for the three water types. Differences among aquaculture samples may reflect uncontrolled factors such as fish density, DNA shedding rate, water circulation, sample volume, extraction efficiency, and potential inhibition. In addition, cellulose acetate filters were used for the relatively low-turbidity RAS samples, whereas mixed cellulose ester filters were used for the other aquaculture and field samples. Because filter material can influence eDNA capture and filtration performance [30,31,32,55], differences among systems cannot be attributed solely to water-matrix characteristics. The aquaculture experiment therefore demonstrates assay application and technical repeatability under the tested conditions rather than complete validation across contrasting environmental matrices.

Several aspects of the field workflow also limit the interpretation of the low-copy detections. Only one 1 L water sample was collected at each station, preventing assessment of within-station heterogeneity and sampling variability, and each field DNA extract was analyzed in only one dPCR reaction. At concentrations near the analytical detection limit, independent water replicates and multiple reactions per extract would improve assessment of repeatability and reduce uncertainty associated with false-positive and false-negative classifications [42,56]. Although the NTCs and field blanks analyzed in this study contained no target-positive partitions, separate extraction-only blanks were not included, limiting the ability to identify contamination potentially introduced specifically during DNA extraction. The filtration apparatus was also pre-rinsed with a small volume of the same sample water before formal filtration to minimize dilution by residual ultrapure water. Although this rinse came from the sample subsequently filtered, introducing sample water into the apparatus before formal filtration complicates strict procedural standardization and should be avoided or independently evaluated in future protocols. Furthermore, field-derived dPCR products were not independently confirmed by sequencing. Sequencing very short and low-copy hydrolysis-probe products can be technically challenging, but molecular confirmation of a subset of environmental detections, where feasible, would strengthen future field applications. Repeated detection from independent water samples and dPCR reactions would provide an alternative source of supporting evidence.

More extensive sampling and analytical replication are needed before this approach can support robust ecological conclusions regarding the distribution or temporal dynamics of wild *B. taipingensis* populations. A single 1 L sample per station may have limited detection probability where target DNA concentrations were very low. Future surveys should evaluate larger filtered volumes and independent biological replicates while considering practical filtration constraints in turbid estuarine water [55,57]. Temporal differences should be assessed through repeated surveys across multiple time points and years rather than two sampling occasions, with the timing and location of stock-enhancement releases explicitly considered in survey design and interpretation. Future analytical work should also include matrix-specific inhibition and recovery assessments, comprehensive field and extraction controls, and replicate dPCR analyses of low-copy samples. Within these limitations, this study provides an initial species-specific dPCR framework for *B. taipingensis* and preliminary field observations that can help guide the design of more highly replicated eDNA monitoring programs for this critically endangered species.

## 5. Conclusions

This study developed a species-specific microfluidic dPCR assay for detecting *B. taipingensis* eDNA and evaluated its analytical performance. The assay showed no cross-amplification with the seven non-target sciaenid species tested and detected and quantified target eDNA in samples from three aquaculture systems, with good technical repeatability under the tested conditions. Preliminary application in the Pearl River Estuary yielded putative target-DNA signals in samples collected during both the April and September 2025 surveys. However, all field detections were below the experimentally established LOQ, and each station was represented by a single 1 L water sample analyzed in a single dPCR reaction. These field observations should therefore be regarded as preliminary evidence of target DNA in the sampled water rather than quantitative estimates of eDNA concentration or abundance, or confirmation that living individuals were present at individual sampling locations. The observed difference between the two surveys may reflect natural seasonal variation, but this cannot be determined from the present data because the potential effects of season cannot be separated from those of the intervening stock-enhancement event and other environmental and sampling differences. The assay provides an initial molecular framework for future eDNA monitoring of *B. taipingensis*, but more robust field application will require independent water replicates, multiple dPCR reactions for low-copy samples, matrix-specific inhibition and recovery assessments, and more extensive temporal sampling. Combining eDNA monitoring with stock-enhancement records, hydrodynamic information, and independent survey approaches could further improve interpretation of future detections and assessment of their possible sources.

## Figures and Tables

**Table 1:** Detection status of *B. taipingensis* eDNA at all field stations in April and September 2025.

	April 2025			September 2025		
Station	*C_rxn_* (Copies µL^−1^)	*C_env_* (Copies L^−1^)	Call	*C_rxn_* (Copies µL^−1^)	*C_env_* (Copies L^−1^)	Call
S1	0.0000	0	ND	0.1075	20	DNQ
S2	0.0000	0	ND	0.0000	0	ND
S3	0.0000	0	ND	0.0000	0	ND
S4	0.0977	18	DNQ	0.0000	0	ND
S5	0.0000	0	ND	0.0000	0	ND
S6	0.0000	0	ND	0.5070	93	DNQ
S7	0.0000	0	ND	0.0000	0	ND
S8	0.0830	15	DNQ	0.0825	15	DNQ
S9	0.0000	0	ND	0.0000	0	ND
S10	0.1608	29	DNQ	0.0811	15	DNQ
S11	0.0000	0	ND	0.0000	0	ND
S12	0.0000	0	ND	0.1838	34	DNQ
S13	0.0000	0	ND	0.3376	62	DNQ
S14	0.0000	0	ND	0.0838	15	DNQ
S15	0.0829	15	DNQ	0.0840	15	DNQ
S16	0.0000	0	ND	0.0000	0	ND
S17	0.0000	0	ND	0.0000	0	ND
S18	0.0000	0	ND	0.0816	15	DNQ
S19	0.0000	0	ND	0.0000	0	ND
S20	0.0000	0	ND	0.0000	0	ND
Z1	0.0000	0	ND	0.0810	15	DNQ
Z2				0.8557	157	DNQ
Z3				0.1635	30	DNQ

Notes: *C_rxn_*, reaction-level concentrations; *C_env_*, environmental concentration; Call, detection call; ND, not detected; DNQ, detected but not quantifiable. Stations Z2 and Z3 were sampled only in September and were excluded from the paired statistical analysis.

## Data Availability

The raw dPCR fluorescence data, partition counts and negative control results that support the findings of this study have been deposited in Zenodo and are publicly available at https://doi.org/10.5281/zenodo.23235851.

## Supplementary Materials

The following supporting information can be downloaded at: https://www.mdpi.com/article/10.3390/biology15201842/s1, Table S1: NCBI BLAST results for *COI* sequences generated from the *B. taipingensis* target specimen and non-target sciaenid specimens requiring molecular confirmation of taxonomic identity; Table S2: Sequences and in silico characteristics of the nine candidate primer pairs evaluated for *B. taipingensis*; Table S3: Reaction-level dPCR output data for samples and controls analyzed in this study; Figure S1: Agarose gel electrophoresis (1.5%) of PCR products amplified from *B. taipingensis* and seven sympatric non-target sciaenid species using nine candidate primer pairs.

## Abbreviations

The following abbreviations are used in this manuscript:

*COI*	Cytochrome *c* Oxidase Subunit I
dPCR	Digital PCR
eDNA	Environmental DNA
LOD	Limit of Detection
LOQ	Limit of Quantification
DNQ	Detected but Not Quantifiable
ND	Not Detected
PRE	Pearl River Estuary
RAS	Recirculating Aquaculture Systems
EP	Earthen Ponds
NMCC	Nearshore Marine Cage-Culture Sites
CA	Cellulose Acetate
MCE	Mixed Cellulose Ester
NTC	No-Template Control
CV	Coefficient of Variation

## Appendix A

### Appendix A.1

This appendix provides the raw reaction-level concentration (*C_rxn_*, copies μL^−1^ per reaction) obtained from eight technical replicates at each of the four low-concentration dilution levels used for LOD and LOQ assessment.

**Table A1:** Raw reaction-level concentration (*C_rxn_*, copies μL^−1^ per reaction) obtained from eight technical replicates at each of the four low-concentration dilution levels used for LOD and LOQ assessment.

Dilution Levels	Replicate 1	Replicate 2	Replicate 3	Replicate 4	Replicate 5	Replicate 6	Replicate 7	Replicate 8
10^−4^	1.58	1.19	1.13	1.38	0.51	1.6	1.86	1.25
4 × 10^−5^	0.167	0.0831	0.169	0.0869	0.268	0.27	0.252	0
2 × 10^−5^	0.0906	0.0948	0.112	0.175	0.093	0.198	0	0
10^−5^	0	0.106	0.0913	0	0.0903	0	0.0827	0

### Appendix A.2

This appendix provides summary statistics for the four low-concentration dilution levels used for LOD and LOQ assessment, including the number of technical replicates, number and detection rate of positive reactions, mean reaction-level concentration (*C_rxn_*, copies μL^−1^ per reaction), standard deviation (SD), coefficient of variation (CV) and exact binomial 95% confidence intervals.

**Table A2:** Summary statistics for the four low-concentration dilution levels used for LOD and LOQ assessment.

Dilution Levels	Technical Replicates	Positive Reactions	Detection Rate (%)	Mean *C_rxn_* (Copies μL^−1^)	SD	CV (%)	Exact 95% CI (%)
10^−4^	8	8	100	1.3125	0.406	30.95	63.06–100
4 × 10^−5^	8	7	87.5	0.162	0.099	61.37	47.35–99.72
2 × 10^−5^	8	6	75	0.095	0.071	74.49	34.91–96.87
10^−5^	8	4	50	0.046	0.050	107.79	15.70–84.30

### Appendix A.3

This appendix provides detailed dPCR quantification data for the three aquaculture water matrices (RAS, EP, and NMCC) used in the assay performance evaluation. The data represent the raw *C_rxn_* values for each sampling point and technical replicate.

**Table A3:** dPCR quantification values (*C_rxn_*, copies μL^−1^ per reaction) for water samples collected from recirculating aquaculture systems (RAS), earthen ponds (EP) and nearshore marine cage-culture sites (NMCC).

Water Matrix	Sampling Points	Replicate 1	Replicate 2	Replicate 3
RAS	F1	737	727	718
RAS	F2	75.9	70.6	76.3
RAS	F3	273	244	271
RAS	F4	312	314	330
RAS	F5	2168	2095	2064
RAS	F6	1843	1881	1855
RAS	F7	108	109	108
RAS	F8	1284	1353	1395
EP	T1	18.1	16	15.7
EP	T2	7.83	7.02	7.64
NMCC	H1	4.06	3.3	3.47
NMCC	H2	55.5	51	53.2
