# Supplementary material for: Searching for a Vanishing Fish: Development of an eDNA-Based Digital PCR Assay and Field Surveys for the Critically Endangered Chinese Bahaba
Source: Biology (Basel). 2026 Oct 14;15(20):1842. doi: 10.3390/biology15201842 (PMC13701327; doi:10.3390/biology15201842)
Supplement: Supplementary file 1 [file biology-15-01842-s001.zip › biology-4557724-supplementary.pdf]

Table S1. NCBI BLAST results for *COI* sequences generated from the *B. taipingensis* target specimen and non-target sciaenid specimens requiring molecular confirmation of taxonomic identity.

Species identified	Closest GenBank BLAST match	Reference accession number	Sequence Identity (%)
Sciaena russelli	*Sciaena russelli*	PZ590380.1	99.85%
Johnius carouna	*Johnius carouna*	NC_035981.1	99.69%
Sciaenops ocellatus	*Sciaenops ocellatus*	OK012052.1	99.69%
Johnius belengerii	*Johnius belengerii*	OM142877.1	99.82%
Nibea albiflora	*Nibea albiflora*	JN242530.1	100.00%
Bahaba taipingensis	*Bahaba taipingensis*	MH260402.1	99.84%

Table S2. Sequences and in silico characteristics of the nine candidate primer pairs evaluated for *B. taipingensis.*

Primer pair	Primer Name	Sequence (5′ – 3′)	Product length （bp）	Tm (°C)	GC (%)
1	F1	CCCTAAGCCTCCTTATCCGG	174	58	60
	R1	GAGCACCAATTATTAGGGGC		55	50
2	F2	CCCTAAGCCTCCTTATCCGG	245	58	60
	R2	CAGGAGCAGAAGGAAGGAAG		57	55
3	F3	TAATAATCGGAGGATTCGGG	170	52	45
	R3	ATAGACCGTCCATCCTGTTC		56	50
4	F4	TAATAATCGGAGGATTCGGG	239	52	45
	R4	AAGGTGTAGAGAGAAGATAG		50	40
5	F5	GCCCCTAATAATTGGTGCTC	144	56	50
	R5	GGAGGATAGACCGTCCATCC		58	60
6	F6	TTGTGCCCCTAATAATTGGT	143	53	40
	R6	ATAGACCGTCCATCCTGTTC		56	50
7	F7	CTTCCTTCCTTCTGCTCCTG	246	56	55
	R7	GGACAGCCCACACAAATAAG		55	50
8	F8	GAACAGGATGGACGGTCTAT	198	55	50
	R8	GGACAGCCCACACAAATAAG		55	50
9	F9	CTATCTTCTCTCTACACCTT	129	59	40
	R9	GGACAGCCCACACAAATAAG		55	50

Note: Primer Tm and GC content were calculated using SnapGene software (version 4.2.4) based on the default nearest-neighbor thermodynamic parameters.

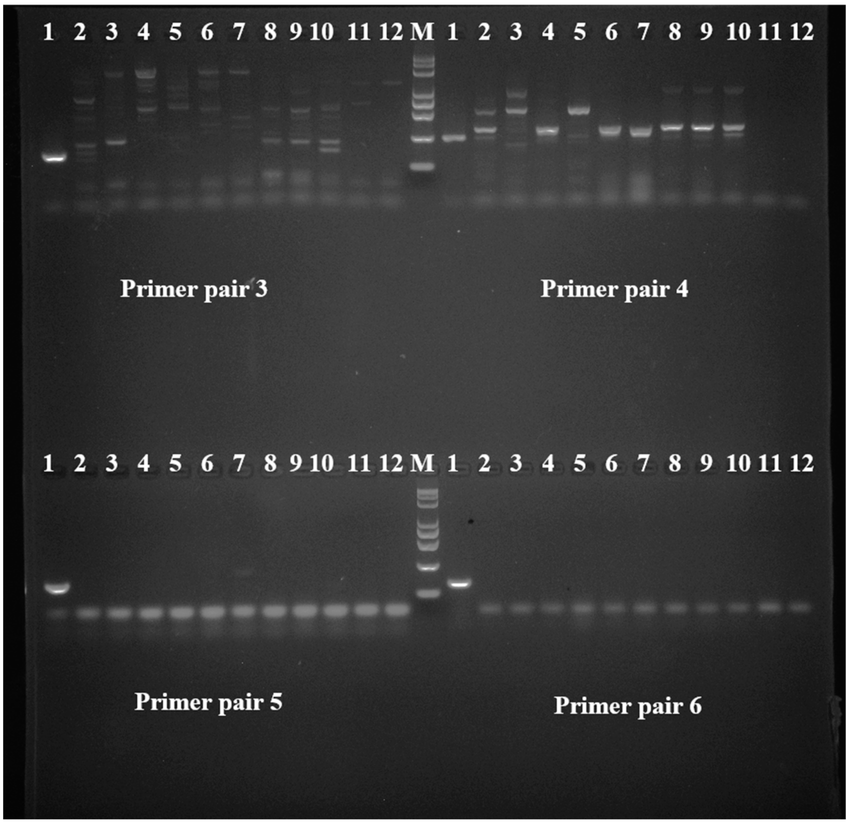
1 2 3 4 5 6 7 8 9 10 11 12 M 1 2 3 4 5 6 7 8 9 10 11 12
Primer pair 3
Primer pair 4
1 2 3 4 5 6 7 8 9 10 11 12 M 1 2 3 4 5 6 7 8 9 10 11 12
Primer pair 5
Primer pair 6

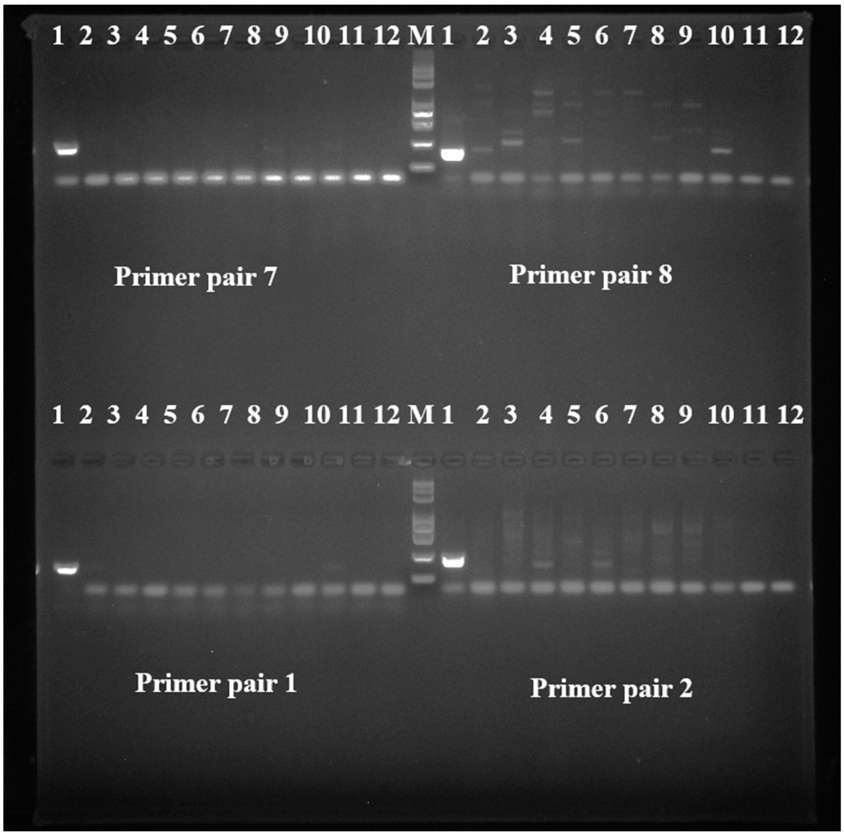
1 2 3 4 5 6 7 8 9 10 11 12 M 1 2 3 4 5 6 7 8 9 10 11 12
Primer pair 7
Primer pair 8
1 2 3 4 5 6 7 8 9 10 11 12 M 1 2 3 4 5 6 7 8 9 10 11 12
Primer pair 1
Primer pair 2

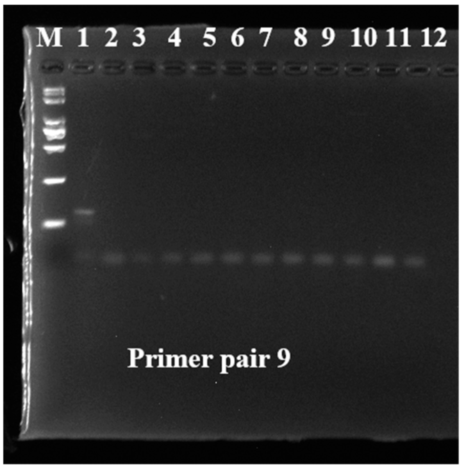

Figure S1. Agarose gel electrophoresis (1.5%) of PCR products amplified from *B. taipingensis* and seven sympatric non-target sciaenid species using nine candidate primer pairs.
Lanes: M, Trans2K Plus DNA Marker; 1. *Bahaba taipingensis*; 2. *Sciaena russelli*; 3. *Collichthys lucidus*; 4. *Johnius carouna*; 5. *Sciaenops ocellatus*; 6. *Johnius belengerii*; 7. *Nibea albiflora*; 8–10. *Nibea miichthioides*; 11. Hood-processed NTC; 12. Aerosol-exposure NTC. The gel image was cropped to remove the portion explicitly identified as unrelated to the present experiment; all experimentally relevant target, non-target, marker, and control lanes were retained.

Table S3. Reaction-level dPCR output data for samples and controls analyzed in this study.

	**Sample Name**	**Status**	**Channel**	**Concentration**	**Positives**	**Negatives**	**Accepted Droplets**	**Threshold**
S.russelli	S1_1	OK	FAM	0	0	19835	19835	1682.66
C.lucidus	S1_2	OK	FAM	0	0	19342	19342	1860.42
J.carouna	S1_3	OK	FAM	0	0	19149	19149	1537.22
S.ocellatus	S1_4	OK	FAM	0	0	19052	19052	1386.73
J.belengerii	S1_5	OK	FAM	0	0	19556	19556	1812.95
N.albiflora	S1_6	OK	FAM	0	0	19663	19663	1533.18
N.miichthioides	S1_7	OK	FAM	0	0	18984	18984	1809.92
NonTarget_mix	neg mix	OK	FAM	0	0	19722	19722	1521.06
*B.taipingensis*_Dil_10^{-7}	S1_9	OK	FAM	0	0	19736	19736	1702.86
*B.taipingensis*_Dil_10^{-6}	S1_10	OK	FAM	0	0	19783	19783	1593.78
*B.taipingensis*_Dil_10^{-5}	S1_11	OK	FAM	0.252338	3	19675	19678	2081.152
*B.taipingensis*_Dil_10^{-4}	S1_12	OK	FAM	1.149052	13	19876	19889	2001.081
*B.taipingensis*_Dil_10^{-3}	S1_13	OK	FAM	10.29485	121	19392	19513	2481.949
*B.taipingensis*_Dil_10^{-2}	S1_14	OK	FAM	107.091113	1222	18281	19503	2675.545
*B.taipingensis*_Dil_10^{-1}	S1_15	OK	FAM	1042.834661	9477	10373	19850	2324.345
Field_Apr_S1	S1	OK	FAM	0	0	21563	21563	1616
Field_Apr_S2	S2	OK	FAM	0	0	20478	20478	1619.03
Field_Apr_S3	S3	OK	FAM	0	0	20052	20052	1754.37
Field_Apr_S4	S4	OK	FAM	0.097659	1	19735	19736	1976.405
Field_Apr_S5	S5	OK	FAM	0	0	6041	6041	1787.7

Field_Apr_S6	S6	OK	FAM	0	0	19709	19709	1568.53
Field_Apr_S7	S7	OK	FAM	0	0	20697	20697	1667.638
Field_Apr_S8	S8	OK	FAM	0.082966	1	19367	19368	1504.979
Field_Apr_S9	S9	OK	FAM	0	0	20463	20463	1534.19
Field_Apr_S10	S10	OK	FAM	0.160751	2	19414	19416	1598.302
Field_Apr_S11	S11	OK	FAM	0	0	20509	20509	1272.889
Field_Apr_S12	S12	OK	FAM	0	0	19224	19224	1502.88
Field_Apr_S13	S13	OK	FAM	0	0	19161	19161	1670.54
Field_Apr_S14	S14	OK	FAM	0	0	19083	19083	1424.1
Field_Apr_S15	S15	OK	FAM	0.082887	1	20572	20573	1571.415
Field_Apr_S16	S16	OK	FAM	0	0	20520	20520	1462.48
Field_Apr_S17	S17	OK	FAM	0	0	20352	20352	1718.425
Field_Apr_S18	S18	OK	FAM	0	0	18781	18781	1825.325
Field_Apr_S19	S19	OK	FAM	0	0	19148	19148	1692.472
Field_Apr_S20	S20	OK	FAM	0	0	18103	18103	1635.19
Field_Apr_Z1	Z1	OK	FAM	0	0	20486	20486	1898.143
Aqua_EP_T2_R1	T2	OK	FAM	7.826889	93	20835	20928	1875.343
Aqua_EP_T2_R2	T2	OK	FAM	7.015259	88	20717	20805	1802.889
Aqua_EP_T2_R3	T2	OK	FAM	7.642785	97	19191	19288	1827.778
Aqua_RAS_F1_R1	F1	OK	FAM	737.295507	6821	14637	21458	1956.373
Aqua_RAS_F1_R2	F1	OK	FAM	727.42815	7181	14005	21186	1628.295
Aqua_RAS_F1_R3	F1	OK	FAM	718.238655	6654	13180	19834	1523.664
Aqua_NMCC_H1_R1	H1	OK	FAM	4.059258	47	19720	19767	1704.253
Aqua_NMCC_H1_R2	H1	OK	FAM	3.301947	42	19261	19303	1564.994
Aqua_NMCC_H1_R3	H1	OK	FAM	3.466414	43	19336	19379	1548.553
Aqua_EP_T1_R1	T1	OK	FAM	18.002667	214	20163	20377	1997.214
Aqua_EP_T1_R2	T1	OK	FAM	15.983644	198	19232	19430	1530.037
Aqua_EP_T1_R3	T1	OK	FAM	15.714845	196	18809	19005	1822.726
Aqua_RAS_F5_R1	F5	OK	FAM	2167.787551	14880	4681	19561	2021.412
Aqua_RAS_F5_R2	F5	OK	FAM	2094.566412	13503	6201	19704	1614.545
Aqua_RAS_F5_R3	F5	OK	FAM	2063.581592	14056	4595	18651	1646.869
Aqua_RAS_F6_R1	F6	OK	FAM	1842.710354	13835	5832	19667	2350.545
Aqua_RAS_F6_R2	F6	OK	FAM	1881.223495	13551	6403	19954	1518.788
Aqua_RAS_F6_R3	F6	OK	FAM	1854.515552	12540	6375	18915	2542
Aqua_RAS_F7_R1	F7	OK	FAM	108.165722	1389	17189	18578	1637.059
Aqua_RAS_F7_R2	F7	OK	FAM	109.299899	1283	18793	20076	1554.879
Aqua_RAS_F7_R3	F7	OK	FAM	107.999765	1293	17510	18803	1804.248
Aqua_RAS_F8_R1	F8	OK	FAM	1283.697003	10674	7046	17720	1418.818
Aqua_RAS_F8_R2	F8	OK	FAM	1353.32453	11192	7113	18305	1673.131
Aqua_RAS_F8_R3	F8	OK	FAM	1395.099458	11753	8134	19887	1550.263
Aqua_RAS_F3_R1	F3	OK	FAM	273.349255	2916	17327	20243	1437.067
Aqua_RAS_F3_R2	F3	OK	FAM	244.252357	2772	17431	20203	2226.111
Aqua_RAS_F3_R3	F3	OK	FAM	271.20727	2944	15510	18454	2247.939
Aqua_RAS_F2_R1	F2	OK	FAM	75.887899	870	19117	19987	1767.691

Aqua_RAS_F2_R2	F2	OK	FAM	70.60983	829	20221	21050	1664.424
Aqua_RAS_F2_R3	F2	OK	FAM	76.334977	883	18149	19032	1546.909
Aqua_RAS_F4_R1	F4	OK	FAM	312.387763	3592	17291	20883	1774.303
Aqua_RAS_F4_R2	F4	OK	FAM	313.621808	3467	17170	20637	1693.495
Aqua_RAS_F4_R3	F4	OK	FAM	329.501056	3552	16663	20215	1912.518
Aqua_NMCC_H2_R1	H2	OK	FAM	55.459591	599	18683	19282	1651.818
Aqua_NMCC_H2_R2	H2	OK	FAM	51.025799	611	18382	18993	1849.616
Aqua_NMCC_H2_R3	H2	OK	FAM	53.236767	622	17923	18545	1556.455
Field_Sep_S1	S1	OK	FAM	0.107456	1	19728	19729	1720.311
Field_Sep_S2	S2	OK	FAM	0	0	19825	19825	1646.3
Field_Sep_S3	S3	OK	FAM	0	0	18566	18566	1729.12
Field_Sep_S4	S4	OK	FAM	0	0	19515	19515	1463.49
Field_Sep_S5	S5	OK	FAM	0	0	17991	17991	1554.39
Field_Sep_S6	S6	OK	FAM	0.507021	6	17936	17942	1485.954
Field_Sep_S7	S7	OK	FAM	0	0	18663	18663	1527.12
Field_Sep_S8	S8	OK	FAM	0.082523	1	20055	20056	2334.923
Field_Sep_S9	S9	OK	FAM	0	0	20217	20217	1502.88
Field_Sep_S10	S10	OK	FAM	0.081093	1	18693	18694	2180.125
Field_Sep_S11	S11	OK	FAM	0	0	19803	19803	1550.35
Field_Sep_S12	S12	OK	FAM	0.183766	2	20333	20335	1838.371
Field_Sep_S13	S13	OK	FAM	0.337642	4	19034	19038	1816.595
Field_Sep_S14	S14	OK	FAM	0.083809	1	19172	19173	2024.078
Field_Sep_S15	S15	OK	FAM	0.083963	1	19137	19138	1928.597
Field_Sep_S16	S16	OK	FAM	0	0	17526	17526	1591.76
Field_Sep_S17	S17	OK	FAM	0	0	19960	19960	1852.34
Field_Sep_S18	S18	OK	FAM	0.081623	1	18046	18047	1989.585
Field_Sep_S19	S19	OK	FAM	0	0	19378	19378	1652.36
Field_Sep_S20	S20	OK	FAM	0	0	17466	17466	2005.86
Field_Sep_Z1	Z1	OK	FAM	0.080967	1	19845	19846	2976.596
Field_Sep_Z2	Z2	OK	FAM	0.855721	14	14625	14639	2413.182
Field_Sep_Z3	Z3	OK	FAM	0.163513	2	19653	19655	1895.152
*B. taipingensis*_Dil_10^{-4}_R1	S1_1	OK	FAM	1.576852	18	19456	19474	1830.333
*B. taipingensis*_Dil_10^{-4}_R2	S1_2	OK	FAM	1.189801	11	18975	18986	1713.131
*B. taipingensis*_Dil_10^{-4}_R3	S1_3	OK	FAM	1.134929	13	16866	16879	1993.018
*B. taipingensis*_Dil_10^{-4}_R4	S1_4	OK	FAM	1.378483	16	19202	19218	1769.576
*B. taipingensis*_Dil_10^{-4}_R5	S1_5	OK	FAM	0.509659	6	18368	18374	1925.818
*B. taipingensis*_Dil_10^{-4}_R6	S1_6	OK	FAM	1.602534	18	17018	17036	2326.636
*B. taipingensis*_Dil_10^{-4}_R7	S1_7	OK	FAM	1.863985	21	17068	17089	1856.606
*B. taipingensis*_Dil_10^{-4}_R8	S1_8	OK	FAM	1.248403	15	17195	17210	1865.455
*B. taipingensis*_Dil_4×10^{-5}_R1	S1_9	OK	FAM	0.167463	2	17591	17593	2009.192
*B. taipingensis*_Dil_4×10^{-5}_R2	S1_10	OK	FAM	0.083059	1	17734	17735	1807.141
*B. taipingensis*_Dil_4×10^{-5}_R3	S1_11	OK	FAM	0.169146	2	17416	17418	1598.566
*B. taipingensis*_Dil_4×10^{-5}_R4	S1_12	OK	FAM	0.086869	1	17450	17451	1883.678
*B. taipingensis*_Dil_4×10^{-5}_R5	S1_13	OK	FAM	0.26799	3	16488	16491	1929.303

*B. taipingensis*_Dil_4×10^{-5}_R6	S1_14	OK	FAM	0.270202	3	16353	16356	2784.222
*B. taipingensis*_Dil_4×10^{-5}_R7	S1_15	OK	FAM	0.252201	3	18561	18564	1908.636
*B. taipingensis*_Dil_4×10^{-5}_R8	S1_16	OK	FAM	0	0	18917	18917	1463.49
*B. taipingensis*_Dil_2×10^{-5}_R1	S1_1	OK	FAM	0.09065	1	18257	18258	1802.588
*B. taipingensis*_Dil_2×10^{-5}_R2	S1_2	OK	FAM	0.094802	1	18537	18538	1797.139
*B. taipingensis*_Dil_2×10^{-5}_R3	S1_3	OK	FAM	0.111828	1	18358	18359	1766.891
*B. taipingensis*_Dil_2×10^{-5}_R4	S1_4	OK	FAM	0.175243	2	16810	16812	1757.566
*B. taipingensis*_Dil_2×10^{-5}_R5	S1_5	OK	FAM	0.093045	1	17269	17270	1822.933
*B. taipingensis*_Dil_2×10^{-5}_R6	S1_6	OK	FAM	0.197928	2	18306	18308	2019.818
*B. taipingensis*_Dil_2×10^{-5}_R7	S1_7	OK	FAM	0	0	17033	17033	1454.4
*B. taipingensis*_Dil_2×10^{-5}_R8	S1_8	OK	FAM	0	0	17661	17661	1612.97
*B. taipingensis*_Dil_10^{-5}_R1	S1_9	OK	FAM	0	0	18387	18387	1323.1
*B. taipingensis*_Dil_10^{-5}_R2	S1_10	OK	FAM	0.106137	1	18738	18739	3199.627
*B. taipingensis*_Dil_10^{-5}_R3	S1_11	OK	FAM	0.091265	1	17098	17099	1939.659
*B. taipingensis*_Dil_10^{-5}_R4	S1_12	OK	FAM	0	0	18040	18040	1418.04
*B. taipingensis*_Dil_10^{-5}_R5	S1_13	OK	FAM	0.090273	1	16792	16793	1862.651
*B. taipingensis*_Dil_10^{-5}_R6	S1_14	OK	FAM	0	0	17991	17991	1466.52
*B. taipingensis*_Dil_10^{-5}_R7	S1_15	OK	FAM	0.082709	1	17809	17810	1788.308
*B. taipingensis*_Dil_10^{-5}_R8	S1_16	OK	FAM	0	0	18672	18672	1403.9
NTC_R1	NTC	OK	FAM	0	0	19477	19477	1729.12
NTC_R2	NTC	OK	FAM	0	0	19604	19604	1419.05
NTC_R3	NTC	OK	FAM	0	0	18866	18866	1490.76
NTC_R4	NTC	OK	FAM	0	0	18197	18197	1361.48
NTC_R5	NTC	OK	FAM	0	0	18044	18044	1368.55
NTC_R6	NTC	OK	FAM	0	0	17524	17524	1521.06
NTC_R7	NTC	OK	FAM	0	0	19058	19058	1557.42
NTC_R8	NTC	OK	FAM	0	0	19033	19033	1765.48
NTC_R9	NTC	OK	FAM	0	0	19257	19257	1735.123
NTC_R10	NTC	OK	FAM	0	0	19144	19144	1552.748
NTC_R11	NTC	OK	FAM	0	0	19575	19575	1934.315
NTC_R12	NTC	OK	FAM	0	0	19748	19748	1787.273
NTC_R13	NTC	OK	FAM	0	0	19019	19019	1742.767
NTC_R14	NTC	OK	FAM	0	0	19639	19639	1786.139
NTC_R15	NTC	OK	FAM	0	0	18453	18453	1411.32
NTC_R16	NTC	OK	FAM	0	0	15001	15001	1369.56
NTC_R17	NTC	OK	FAM	0	0	18219	18219	1616.565
NTC_R18	NTC	OK	FAM	0	0	17322	17322	1373.856
NTC_R19	NTC	OK	FAM	0	0	18335	18335	1202.91
NTC_R20	NTC	OK	FAM	0	0	20673	20673	1483.69
Filed-Apri-blank-R1	blank	OK	FAM	0	0	20836	20836	1794.77
Filed-Apri-blank-R2	blank	OK	FAM	0	0	19496	19496	1633.17
Filed-Apri-blank-R3	blank	OK	FAM	0	0	20457	20457	1482.68
Filed-Apri-blank-R4	blank	OK	FAM	0	0	20751	20751	1769.507
Filed-Apri-blank-R5	blank	OK	FAM	0	0	19777	19777	1571.56

Filed-Sep-blank-R1	blank	OK	FAM	0	0	18518	18518	1186.75
Filed-Sep-blank-R2	blank	OK	FAM	0	0	19897	19897	1773.947
Filed-Sep-blank-R3	blank	OK	FAM	0	0	19591	19591	1955.844
Filed-Sep-blank-R4	blank	OK	FAM	0	0	20790	20790	1258.46
Filed-Sep-blank-R5	blank	OK	FAM	0	0	19765	19765	1250.38
B. taipingensis	muscle	OK	FAM	23.380205	195	14560	14755	1486.101
B. taipingensis	muscle	OK	FAM	24.900481	311	19335	19646	2066.171
B. taipingensis	muscle	OK	FAM	26.37475	329	20481	20810	1554.091
B. taipingensis	Muscle	OK	FAM	41.355333	524	17880	18404	1584.667
B. taipingensis	Muscle	OK	FAM	42.425601	510	19062	19572	1552.727
